# The t(14;18) translocation is absent from endothelial and follicular dendritic cells of follicular lymphoma (FL) and shows heterogeneous presence in preserved FL mantle zones

**DOI:** 10.1186/s13000-018-0703-2

**Published:** 2018-05-02

**Authors:** Perikles Kosmidis, Barbara Mankel, Falko Fend, Patrick Adam

**Affiliations:** Institute of Pathology and Neuropathology and Comprehensive Cancer Center (CCC), Eberhard-Karls-University Tübingen, Liebermeisterstr. 8, 72076 Tübingen, Germany

**Keywords:** T(14;18), Microenvironment cells, Follicular lymphoma

## Abstract

**Background:**

The translocation t(14;18)(q32;q21) is the genetic hallmark of follicular lymphoma (FL) and can be observed in 85–90% of cases. Whether the translocation is restricted to cells with germinal center B-cell phenotype or can be observed in other cell types of the microenvironment remains debated. Of interest, cases of associated histiocytic and dendritic cell sarcomas arising in the background of FL have been shown to be clonally related and carry the t(14;18), suggesting a “transdifferentiation” of the malignant FL clone into a neoplasm of a different hematopoietic lineage.

**Methods:**

We analyzed the presence of the t(14;18)(q32;q21) as a surrogate marker of the malignant clone in cells of the FL microenvironment using combined fluorescence immunophenotyping and interphase cytogenetics targeting the *BCL2* gene locus. In addition to non-lymphoid cells in FL, we analysed FL with preserved IgD+ mantle zones and cases of in situ follicular neoplasia (ISFN) to investigate whether cells of non-germinal center B-cell phenotype are part of the malignant clone.

**Results:**

Six (40%) of 15 manifest FL cases with preserved IgD+ mantle zones did not harbour the t(14;18)(q32;q21) translocation. In all t(14;18) + FL cases, follicular dendritic cells and endothelial cells lacked the t(14;18) translocation. 2/9 FL revealed t(14;18)- IgD+ mantle zone B-cells. In the seven ISFN cases, the t(14;18) translocation was strictly confined to germinal center cells.

**Conclusions:**

The t(14;18) translocation in follicular lymphoma is limited to B-cells. The origin of IgD+ mantle cells is heterogeneous, in the majority of cases belonging to the neoplastic clone, whereas a minority of cases of manifest FL show nonneoplastic mantle zones, similar to ISFN.

## Background

Follicular lymphoma (FL) is characterized in the majority of cases by the recurrent chromosomal translocation t(14;18)(q32;q21), which brings the *BCL2* gene on chromosome 18 under the influence of the *IgH* promoter on chromosome 14, resulting in a constitutive overexpression of the antiapoptotic BCL2 protein [1]. Although this genetic hallmark of FL is known for decades, it is still a matter of debate whether the translocation resides in cells other than the neoplastic population with a germinal center B-cell phenotype.

The observation of non-lymphoid neoplasms, e.g. tumors of dendritic cells occurring in patients with follicular lymphoma sharing the chromosomal translocation t(14;18)(q32;q21), the genetic hallmark of FL, suggested a “transdifferentiation” of the malignant FL clone into a neoplasm of a different lineage or origin of the two neoplasms from a single precursor cell [2]. This and subsequent observations provided evidence for a common clonal origin of FL and dendritic cell neoplasms and it has been concluded that some sort of “lineage plasticity” may also occur in mature lymphoid neoplasms. This concept was further supported by the observation of clonal gene rearrangements of the immunoglobulin heavy or light chain gene in nearly half of the cases of sporadic histiocytic/dendritic cell sarcomas [3]. In the same study, one of the 23 cases analyzed, also harbored the t(14;18).

Also in the setting of a transformation of FL into a diffuse large B-cell lymphoma (DLBCL) repeatedly a syn- or metachronous manifestation of a histiocytic / dendritic cell sarcoma (H/DCS) has been observed [4]. Again, in these cases the t(14;18) could be detected in the DLBCL, in the H/DCS transformed cell population, as well as in the underlying FL component. Interestingly, very recently, the spectrum of transdifferentiation has been expanded again by the observation of two cases of clonally related FL and Langerhans cell neoplasms [5]. Also in these cases, immunoglobulin gene and *BCL2* rearrangement analyses confirmed the clonal relationship between the FL and the Langerhans cell neoplasm.

An alternative explanation for transdifferentation is the origin of these neoplasms from a common pluripontent progenitor cell. In a study of B-cell lymphomas, among them 14 FL, the lymphoma-specific recurrent genetic imbalances were detected by FISH in the endothelial cells of the tumor-associated vascular structures [6]. In all of the 14 FL cases, the t(14;18)(q32;q21) and in some cases further aberrations like trisomy 5 and/or 7 were detected with an incidence rate between 18 and 80% in endothelial cells [6]. Although cytogenetic abnormalities in tumor-associated endothelial cells have also been reported for other tumor types, the occurrence of lymphoma-specific translocations in stromal cells is still controversial [7, 8].

A related question is, whether the t(14;18) translocation occurs in FL only in B-cells of germinal center phenotype or can also be identified in other B-cells within involved nodes. In the earliest morphological detectable stage of FL, the so-called “*in situ* follicular neoplasia” (ISFN), defined as immunohistochemically detectable strongly BCL2 expressing B-cells within the germinal center structures of otherwise reactive lymph nodes, it is assumed that only the BCL2+ GC cells carry the translocation. Therefore, the aims of this study were 1) to determine the presence of the t(14,18) in the adjacent mantle zone of ISFN and manifest FL with detectable IgD+ mantle zone cells, and 2) to analyze the presence of the t(14;18) in endothelial and follicular dendritic cells of manifest FL using combined immunofluorescence and FISH (FICTION).

## Methods

### Case selection

Sixteen FL cases (WHO grade 1/2) with intact mantle zones identifiable in routine H&E staining and seven ISFN cases from the archives of the Institute of Pathology of the University of Tübingen, Germany, from which sufficient tissue was available, were included in the study. Cases were re-evaluated using H&E and Giemsa stains and standard diagnostic immunohistochemistry. The grading of FL was performed following the recommendations of the 2016 update World Health Organization (WHO) classification of tumours of haematopoietic and lymphoid tissues [1]. The study was approved by the local ethics committee.

### Immunohistochemical analysis

All FL cases were stained for CD20 (DAKO, Hamburg, Germany; dilution 1:500), CD10, CD23 (both Novocastra, Berlin, Germany; both dilution 1:30) CD3 (DCS, Hamburg, Germany; dilution 1:100), BCL6 (Zytomed, Berlin, Germany; dilution 1:25), MIB1 (Ki67; DAKO, Hamburg, Germany; dilution 1:200) and BCL2 (clone 100/D5 against residues aa 41–54; DAKO, Hamburg, Germany; dilution 1:50). Cases that were negative in the staining with BCL2 (100/D5) were additionally stained with the two alternative BCL2 antibodies clone E17 (Zytomed, Berlin, Germany; dilution 1:50, against residues aa 61–76) and SP66 (Cell Marque, Rocklin, California, USA; dilution 1:100, against the N-terminal portion of the protein). Cases of “*in situ* follicular neoplasia” (ISFN) were stained with BCL2 (100/D5) and MIB1. Immunohistochemical staining was performed on formalin-fixed, paraffin-embedded tissue sections on an automated immunostainer (Ventana Medical Systems©, Tucson AZ, USA) following the manufacturer’s protocols.

### Fluorescence in situ hybridization (FISH)

In all FL and ISFN cases, the presence of the chromosomal translocation t(14;18) (q32;q21) was analyzed by fluorescence in situ hybridization (FISH), using a break-apart probe for the *BCL2* gene locus (Vysis LSI *BCL2* Dual Color Break Apart Rearrangement Probe, Abbott Molecular, Wiesbaden, Germany), according to manufacturer’s instructions. A detailed description of the FISH and data analysis is given in the FICTION method part below.

### Fluorescence immunophenotyping and interphase cytogenetics as a tool for the investigation of neoplasms (FICTION)

In this study, FICTION, used as a combination of immunohistochemistry for the cell typing and FISH (fluorescence in situ hybridization) for the detection of the t(14;18), was carried out on 4-μm-thick paraffin sections. For the identification of mantle zone B-cells, endothelial cells and follicular dendritic cells, we used IgD (DAKO, FLEX polyclonal Rabbit Anti-Human IgD, Cat.-No.: A0093), CD34 (Clone DBEnd-10, DAKO Denmark, Code M7165), and clusterin antibodies (Apolipoprotein J) (Novocastra™/LEICA MICROSYSTEMS, Newcastle, UK).

Paraffin sections were de-waxed (3× times for 10 min in Xylene) and rehydrated (2× times for 5 min in ethanol). Endogenous peroxidase activity was blocked using 3% H_2_O_2_ for 10 min. After antigen retrieval by pressure cooking in sodium citrate buffer (pH 6.0) for 3.5 min and rinsing with TBS (pH 7.4), the slides were incubated for 3 min at 37 °C with 250 μl/ml Pepsin (Sigma-Aldrich/Merck, Sant-Louis/Missouri, Cat. No.: P6887, Pepsin stock solution 25 mg/ml, pepsin final working solution 250 μg/ml diluted in 37 °C pre-heated sodium chloride). After treatment with blocking solution for 15 min primary antibody incubation for 60 min at room temperature was performed. All antibody mixes, including the secondary antibodies, were diluted with the ready to use Antibody Diluent from DAKO/Agilent Technologies (Cat. No. S0809).

For IgD staining, a dilution of 1: 400 was used for the preparation of the antibody mix and a total volume of 800 μl was applicated per slide. Thus, 2 μl of IgD antibody plus 798 μl of antibody diluent were used for the IgD mix.

For the clusterin staining a dilution of 1: 30, for the CD34 staining a dilution of 1: 50 was used. The secondary antibodies were labelled with Cy™2 and Cy™3 antibodies. The dilution of the Rabbit Anti-Mouse Cy™3 antibody was 1: 25 and the Goat Anti-Mouse Cy™2 and Cy™3-labelled antibodies were used in a 1: 50 dilution (Table 1).

**Table 1:** Primary and secondary antibodies for the FICTION method

Primary Ab	Source	Dilution	Incubation Time	Secondary Ab	Source	Dilution	Incubation Time	Conjugated with
Polyclonal Ab Rabbit anti-Human IgD Code: A0093	DAKO/Agilent Technolo.	1:400	60 min/RT	Goat anti Rabbit IgG (H + L)	Jackson ImmunoResearch (Dianova distrib.)	1:25	60 min/RT	Cy™3, Code:111–165-144
mAb Mouse anti-Human CD34 Class II Clone QBEnd-10 Code: M7165	DAKO/Agilent Technolo.	1:50	60 min/RT	Goat anti Mouse IgG (H + L)	Jackson ImmunoResearch (Dianova distrib.)	1:50 1:25 1:50	60 min/RT	Cy™2, Code:115–228-003 Cy™3, Code:115–165-003 Alexa Flour^ʘ^ 488, Code: 115–545-003
mAb Mouse anti-Human IgG1 kappa Clusterin (Apolipoprotein J) Code: NCL-CLUSTERIN	Novocastra (LEICA distribut.)	1:30	60 min/RT	Goat anti Mouse IgG (H + L)	Jackson ImmunoResearch (Dianova distrib.)	1:50 1:25 1:50	60 min/RT	Cy™2, Code:115–228-003 Cy™3, Code:115–165-003 Alexa Flour^ʘ^ 488, Code: 115–545-003

After incubation of the secondary antibody, the slides were washed in graded alcohol series and dried on air. Before the application of the Vysis LSI BCl2 dual color break apart rearrangement probe (Abbott Molecular/Vysis, Illinois/USA, Cat. No.: 05 N51–020) was denatured for 5 min at 73 °C. After applying the probe and sealing the coverglass with Fixogum, a rubber cement special adhesive (Fixogum, Tamm/Germany, Cat. No. 290117000), the mixture was incubated in a humid chamber (ThermoBrite from Abbott Molecular/Vysis, Cat. No. 07 J91–020) for 17 h at 37 °C. On the second day, adhesive and coverglass were carefully removed and the slides were washed with 2xSSC at room temperature for 5 min. After an additional wash step with the detergent 0.3% NP-40 at 73 °C in a 2xSSC solution, a final washing with pure 2xSSX was carried out for 5 min to remove the non-(specific)-bound probe. After quick dehydration series in ethanol and air-dried the fluorescence dye DAPI (4′, 6-diamidine-2-phenylindole, component of the BCl2 probe kit from Abbott/Vysis) was used for counter-staining.

Appropriate positive controls from FL and negative controls from hyperplastic tonsils were evaluated simultaneously. In a translocation negative cell the expected signal pattern is two orange/green (yellow) fusion signals. Translocation positive cells showed one separated orange and green signal pair in addition to an intact fusion signal of the second allele.

The cut-off value for the presence of the t(14;18) was calculated with 10%. The evaluation of the signals was carried out using a Zeiss Axio Scope A1 microscope (HXP 120C) (Zeiss, Oberkochen, Germany). The fluorescence filters used for the two signals from the FISH for spectrum green and orange were Cyanine Cy™2 (excitation = 493 nm / emission = 519 nm), Indocarbocyanine Cy™3 dye (excitation = 550 nm / emission = 570 nm) and DAPI (excitation = 350 nm / emission = 470 nm).

For the evaluation as a first step the intensity of the immunostaining of the respective case was compared to the controls. Several high power fields (600× magnification) were screened and the IgD-, CD34- and clusterin-positive cells, respectively were evaluated. A total of at least 100 positively stained cells containing both signal types and a DAPI stained cell nucleus were evaluated per case. Analyses of all cases were performed in triplicates.

For photo documentation the supplied Zeiss software and the Zeiss Monochromator camera (Zeiss, Oberkochen, Germany) were used.

## Results

**Fig. 1:**
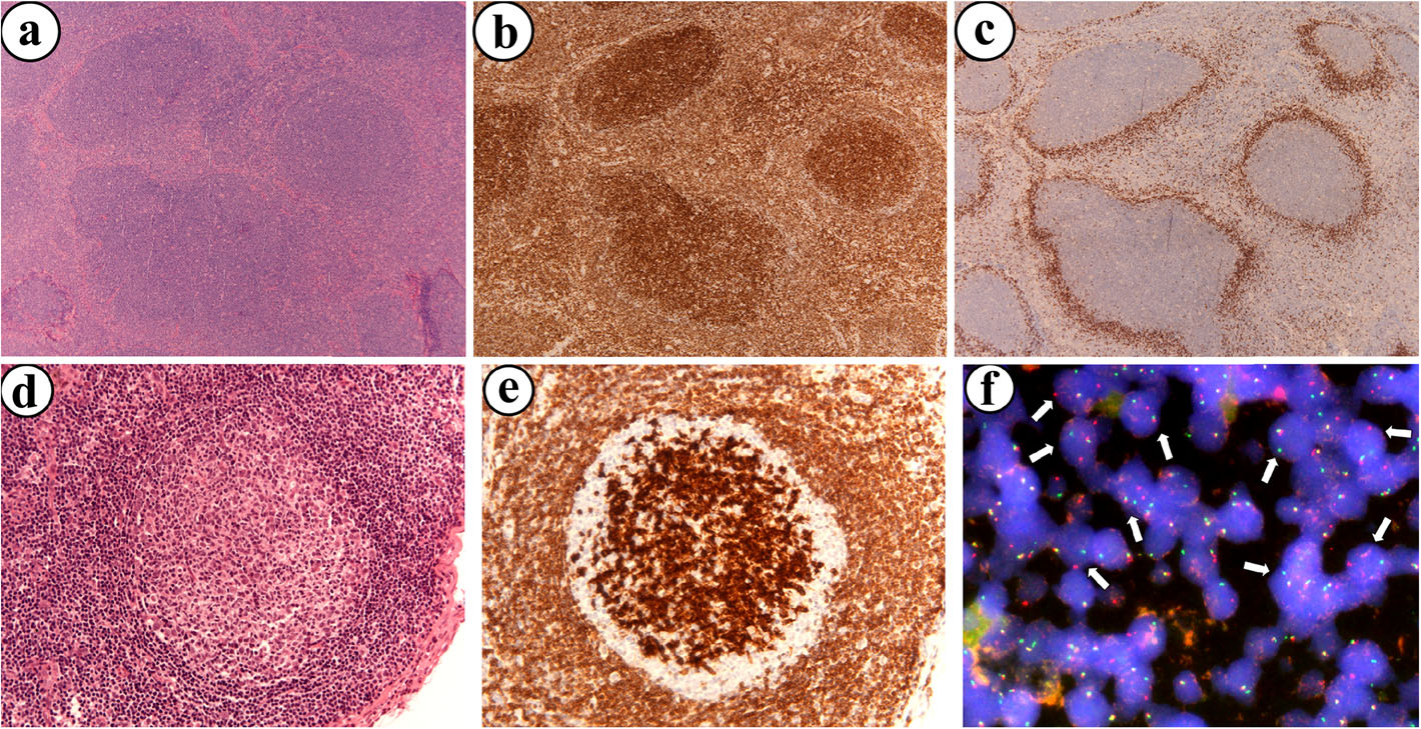
Follicular lymphoma case for representative demonstration (**a**-**c**) with H&E staining (**a**), BCl2 (**b**) and IgD (**c**) staining, respectively (Original magnification × 400). Representative morphologic and immunohistochemical findings of the in situ neoplasia case (**c**-**e**): Haematoxylin & Eosin (H&E) staining (**d**) and BCL2 immunohistochemistry (**e**) (Original magnification × 400). The chromosomal translocation t(14;18) was detectable in the in situ neoplasia cells using the FICTION technique (**f**) (Original magnification × 1000)

Fifteen cases of FL grade 1/2 with a predominant follicular growth pattern and morphologically and phenotypically detectable IgD+ mantle zone structures without IgD expression in the neoplastic germinal centers were included in the analysis (Fig. 1a-c). Of these 15 cases, 6 (=40%) cases were immunohistochemically negative for BCL2 and did not harbour the t(14;18) in the FISH analysis. The remaining 9 FL cases and seven additional cases of “in situ” follicular neoplasia (ISFN) are the group of interest in this study. FISH analysis using a *BCL2* break-apart probe demonstrated a *BCL2* break in these 9 cases and in all seven ISFN cases. The cases had the characteristic FL phenotype with positivity for CD20, CD10 and BCL6. CD23 staining showed only the disrupted networks of follicular dendritic cells but was negative in the tumor cells.

### FICTION analysis of mantle zones in “in situ” follicular neoplasia (ISFN)

All seven ISFN cases showed clearly detectable breaks in the *BCL2* gene locus in the FISH analysis (Fig. 1f). These breakage events were strictly confined to the neoplastic cells inside the germinal center structures and matched the pattern of distribution of immunohistochemically strongly BCL2 protein expressing B-cells (Fig. 1e). The cells of the mantle zone only showed fused signals, indicating intact *BCL2* gene loci without evidence of a t(14;18).

### FICTION analysis of t(14;18) + positive FL cases with preserved mantle zones

**Fig. 2:**
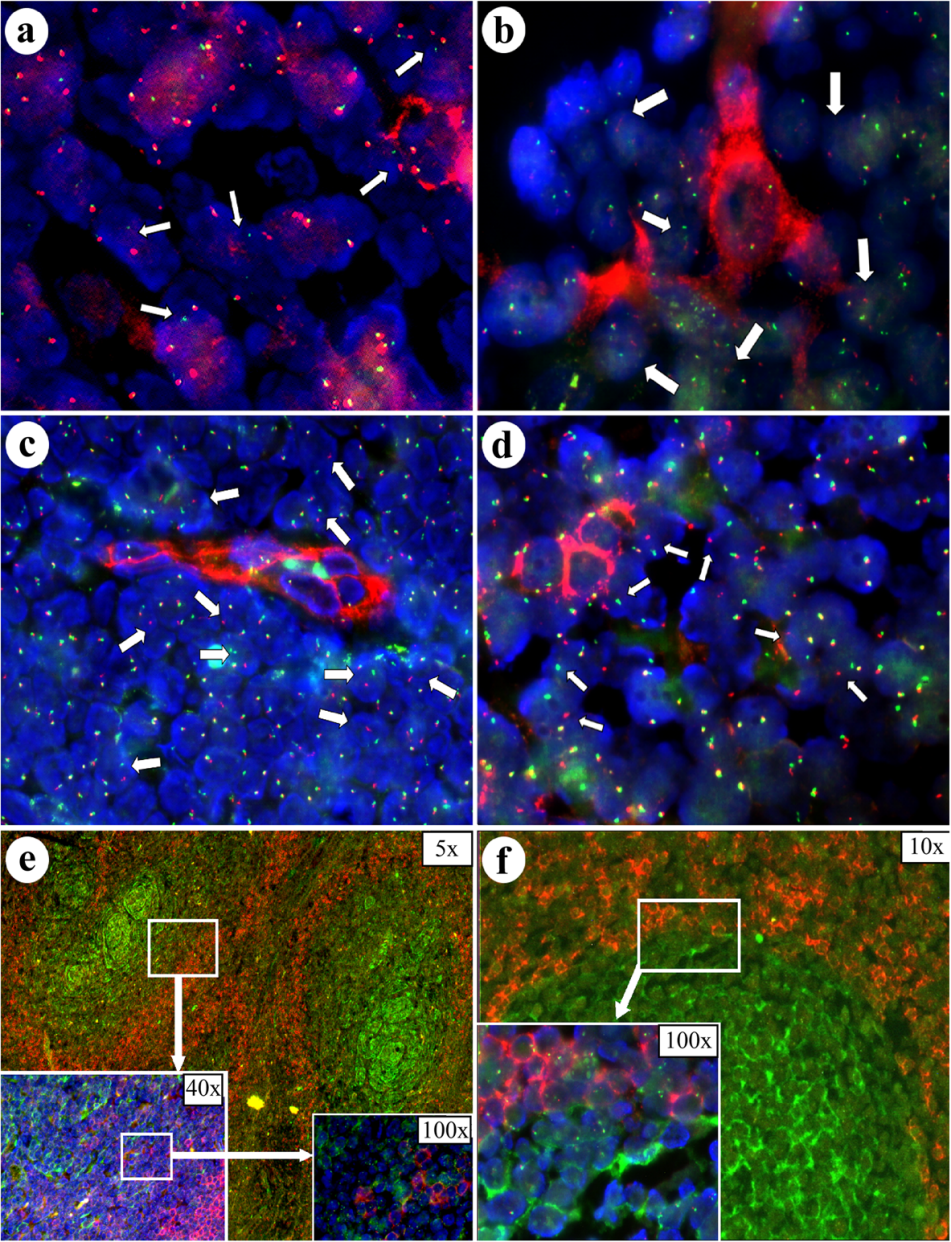
**a**, **b** FICTION technique: FISH method in combination with immunohistochemical IgD staining. Well identifiable hybridization signals in the IgD + cells as well as in the IgD - neighborhood, here with t(14;18) translocations (arrows) (Original magnification × 1000 (**a**,**b)**. **c** FICTION with t(14;18) transcolations (arrows) in the vicinity of single or **d** clusters of CD34 stained cells, each without translocation t(14;18) (Original magnification × 600 (**c**,**d)**). **e**, **f** Combined FICTION method of simultaneously stained IgD (red) and clusterin (green) cells (**f**) (Original magnification: × 200/× 1000 (**e**,**f)** each). Arrows show translocated signals

The FICTION analysis allowed a very precise definition of IgD positive mantle zone cells. These cells showed a split with the dual color *BCL2* gene locus probe in seven of the nine cases evaluated (=78%), indicating a *BCL2* gene break in these cells as a surrogate marker for the t(14;18) (Fig. 2a, b). In contrast, in the remaining two cases IgD+ mantle cells showed only fused signal pairs, thus indicating intact *BCL2* gene loci in contrast to the IgD- B-cells of the neoplastic germinal centers.

### Absence of BCL breaks in non-lymphoid cells of FL

CD34 staining decorated endothelial structures of which mostly the small calibre vessels (high endothelial venules) were evaluable. In all cases analysed, these cells did not show any breaks in the *BCL2* gene locus (Fig. 2c, d).

Staining for clusterin allowed for the identification of follicular dendritic cells. Again, none of these cells showed a signal splitting indicative for the presence of the t(14;18) translocation (Fig. 2e, f).

## Discussion

The presence of the t(14;18) translocation in non-lymphoid cells in FL has been described more than a decade ago, using a similar technical approach as our study [6]. This finding, however, has to our knowledge not been addressed further by other groups. The more recent description of clonally related cases of histiocytic and dendritic cell neoplasms in patients with follicular lymphoma sharing the t(14;18) translocation [2, 5, 9] has prompted us to re-evaluate the presence of the t(14;18) translocation in non-lymphoid cells of the microenvironment in FL. Our study indicates that the t(14;18) translocation is confined to B-cells and usually does not occur in stromal cells of involved lymph nodes. In addition, we demonstrate that IgD+ follicle mantle cells in a minority of manifest FL cases are non-neoplastic B-cells, similar to the situation in ISFN.

Using the FICTION approach we were able to simultaneously identify the lineage of the respective cells by fluorescence immunohistochemistry and the presence of a break in the *BCL2* gene locus, indicative for a t(14;18) by fluorescence in situ hybridization (FISH) [10, 11]. FICTION is a powerful technique [12–14], which has been used extensively in haematopoietic malignancies, e.g. to identify BCL6 imbalances in nodular lymphocyte predominant Hodgkin lymphoma [15] or 14q32 chromosomal rearrangements in multiple myeloma [16].

Given the fact that several cases of FDC sarcomas with t(14;18) translocation in FL patients have been described, we used clusterin as selective and effective marker for FDCs [11] in our analysis. While the surrounding FL cells clearly showed the characteristic split signals, the clusterin positive follicular dendritic cells only harboured fused signal pairs, indicating intact *BCL2* gene loci. This finding indicates that FDC are not normally part of the malignant clone but are recruited by the neoplastic germinal center B-cells. For cases with clonally related dendritic cell neoplasms, one can hypothesize that either true transdifferentiation occurs, or that both FDC sarcoma and FL arise from a common progenitor cell carrying the t(14;18) translocation.

In the study mentioned above, not only dendritic cells, but also endothelial cells were found to carry the t(14;18) translocation in all of the 14 included FL cases, with a frequency between 18 and 80% of cells [6]. However, using FICTION with an anti-CD34 staining to detect endothelial cells within the neoplastic follicles, we again were not able to identify *BCL2* gene locus breaks in these cells. The reason for this discrepancy is not clear, but case selection or technical aspects such as superimposed B-cells or section artefacts may play a role.

A second question addressed in our study was the extent to which B-cells with a non-germinal center phenotype in FL are part of the malignant clone. In order to select cases with clearly discernible separate B-cell populations, we identified cases of manifest FL with partially preserved IgD+ follicle mantle zones. As controls, we used cases of ISFN, which are regarded as a precursor lesion consisting of t(14;18)+, BCL2-expressing B-cells restricted to germinal centers of morphologically reactive lymph nodes. In concordance with this definition, we did not find B-cells with BCL2 breaks in the mantle zones of the 7 cases of ISFN studied in our series.

Of interest, 6 of 15 (40%) FL cases with morphologically intact IgD+ mantle zones were immunohistochemically negative for the BCL2 protein and also did not harbour the t(14;18) in the FISH analysis, a negativity rate higher than expected for conventional FL grade 1/2, with a 10–15% reported in the literature [10], although we took care to exclude FL mimics, such as nodal marginal zone lymphoma. In the remaining 9 cases of manifest FL with t(14;18) translocation, the FICTION analysis allowed a precise identification of the mantle zone cells by their distinctive IgD positivity in the immunofluorescence staining (Fig. 2a, b). In 7 of 9 cases, the IgD+ mantle zone cells harboured a t(14;18) and therefore were part of the tumor clone.

In two of nine IgD+ cases the cells of the mante zone only showed fused signal pairs, thus indicating intact *BCL2* gene loci without evidence of a t(14;18), similar to the situation on ISFN. Although there was no obvious morphological or phenotypical difference between the cases with t(14;18)-positive and negative mantle zone cells, our findings indicate that both mantle zone B-cell-like differentiation, as well as preservation of pre-existent non-neoplastic mantle zones can occur in FL.

## Conclusions

In summary, our data do not provide evidence for the frequent presence of the t(14;18) in non-lymphoid cells of FL. Whereas mantle zone cells in ISFN usually are reactive B-cells, and the BCL2 translocation is restricted to germinal center B-cells, IgD+ mantle cells in manifest FL usually, but not always are part of the malignant clone.

## Abbreviations

DAPI: 4′,6-diamidino-2-phenylindole
DLBCL: Diffuse large B-cell lymphoma
FDC: Follicular dendritic cell
FICTION: Fluorescence immunophenotyping and interphase cytogenetics as a tool for the investigation of neoplasms
FISH: Fluorescence in situ hybridization
FL: Follicular lymphoma
H/DCS: Histiocytic / dendritic cell sarcoma
ISFN: In situ follicular neoplasia
